# Sodium-mediated plateau potentials in an identified decisional neuron contribute to feeding-related motor pattern genesis in *Aplysia*

**DOI:** 10.3389/fncir.2023.1200902

**Published:** 2023-06-09

**Authors:** Alexis Bédécarrats, John Simmers, Romuald Nargeot

**Affiliations:** CNRS, UMR 5287, Institut de Neurosciences Cognitives et Intégratives d’Aquitaine, University of Bordeaux, Bordeaux, France

**Keywords:** feeding, *Aplysia*, plateau potential, B63, central pattern generator

## Abstract

Motivated behaviors such as feeding depend on the functional properties of decision neurons to provide the flexibility required for behavioral adaptation. Here, we analyzed the ionic basis of the endogenous membrane properties of an identified decision neuron (B63) that drive radula biting cycles underlying food-seeking behavior in *Aplysia*. Each spontaneous bite cycle arises from the irregular triggering of a plateau-like potential and resultant bursting by rhythmic subthreshold oscillations in B63’s membrane potential. In isolated buccal ganglion preparations, and after synaptic isolation, the expression of B63’s plateau potentials persisted after removal of extracellular calcium, but was completely suppressed in a tetrodotoxin (TTX)- containing bath solution, thereby indicating the contribution of a transmembrane Na^+^ influx. Potassium outward efflux through tetraethylammonium (TEA)- and calcium-sensitive channels was found to contribute to each plateau’s active termination. This intrinsic plateauing capability, in contrast to B63’s membrane potential oscillation, was blocked by the calcium-activated non-specific cationic current (*I*_*CAN*_) blocker flufenamic acid (FFA). Conversely, the SERCA blocker cyclopianozic acid (CPA), which abolished the neuron’s oscillation, did not prevent the expression of experimentally evoked plateau potentials. These results therefore indicate that the dynamic properties of the decision neuron B63 rely on two distinct mechanisms involving different sub-populations of ionic conductances.

## New and noteworthy

Here, we report an endogenous plateau property underlying bursting in a bilateral pair of buccal ganglion pacemaker neurons (B63) which trigger individual motor pattern cycles for food-seeking behavior in the marine mollusk *Aplysia.* The ionic mechanisms of this membrane bistability in B63 rely critically on voltage-dependent sodium inward currents. The expression of the plateauing property and the underlying endogenous oscillatory pacemaker drive can be dissociated pharmacologically, indicating that the two intrinsic properties depend on different sets of conductances. Our results thus shed new light on the mechanisms of food-seeking decision-making in *Aplysia*.

## Introduction

Bistable membrane behavior giving rise to depolarized, burst-generating plateau potentials is a widespread endogenous membrane property of neurons in central pattern generating (CPG) networks for diverse rhythmic behaviors such as breathing, chewing or walking (Russell and Hartline, 1978; Hounsgaard et al., 1984; Kiehn, 1991; Onimaru et al., 1996; Russo and Hounsgaard, 1996; Brocard et al., 2006; Husch et al., 2015; Boeri et al., 2018). Although many neurons are capable of action potential bursting, the contribution of an underlying plateau potential capability has not always been established.

Food-seeking behavior in *Aplysia* partly consists of cycles of protraction/retraction of its tongue-like radula which are triggered by decision neurons located in the buccal ganglia (Cropper et al., 2004; Nargeot and Simmers, 2011). The decision-making process responsible for the expression of buccal motor pattern cycles (BMPs) is based on intracellular calcium release-derived oscillations in membrane potential of two bilateral pacemaker neurons, B63, and their excitatory synaptic connections with electrically coupled neurons, including the B31/B32 motor neurons, in the buccal CPG network (Susswein et al., 2002; Bédécarrats et al., 2021). In contrast, earlier experimental and computational data suggested that the B63 depolarizations producing the impulse bursts that drive individual radula bite cycles may not rely on an intrinsic property of this neuron *per se*. Rather, they were proposed to result from B63’s fast cholinergic synaptic excitation of the B31/B32 neurons and a prolongation of the latters’ depolarizing response and associated firing by voltage-activated autapses. This in turn provides depolarizing-sustaining feedback excitation of B63 through electrical coupling (Susswein et al., 2002; Cataldo et al., 2006; Baxter et al., 2010; Costa et al., 2020; Momohara et al., 2022). However, we recently found that B63 can express spontaneous or experimentally evoked plateau potentials in modified bathing saline that blocks chemical synapses in the buccal network (Bédécarrats et al., 2021). In the present study we now provide evidence for the ability of B63 to produce plateau potentials independently of B31/B32 depolarization under standard physiological conditions. We then characterize the ionic basis of these B63 plateau potentials under complete chemical synapse blockade. Our results therefore suggest that in addition to the previously described synaptic mechanism for plateau-like behavior, the B63 pacemaker neurons play an active role in the decision-making process not only through their endogenous oscillatory property, but also via a distinct intrinsic plateauing capability.

## Methods

### Animals

*Aplysia californica* (6–10 months old) purchased from the University of Florida were housed in tanks containing fresh aerated sea water (∼15°C) and fed with seaweed (*Ulva lactuca*) obtained from the Station Biologique at Roscoff, France.

### Isolated buccal ganglia preparations

Animals were anesthetized with an injection of a 60 ml isotonic MgCl_2_ solution (in mM: 360 MgCl_2_, 10 HEPES, adjusted to pH 7.5). Buccal ganglia were dissected under artificial sea water (ASW, in mM: 450 NaCl, 10 KCl, 30 MgCl_2_, 20 MgSO_4_, 10 CaCl_2_, 10 HEPES, adjusted to pH 7.5). The isolated ganglion preparations were maintained at 15°C with a Peltier cooling device and bathed in standard ASW or modified saline during subsequent pharmacological experiments.

### Modified saline and pharmacology

A “Low Ca + Co” solution was used to block all network chemical synaptic connections and was composed of: (in mM) 446 NaCl, 10 KCl, 30 MgCl_2_, 20 MgSO_4_, 3 CaCl_2_, 10 CoCl_2_, 10 HEPES, adjusted to pH 7.5. The NaCl concentration was adjusted to maintain the same osmolarity as ASW. Electrophysiological recordings under this saline started at least 20 min after perfusion onset, which was the time required to completely block chemical synapses and allow for recovery of neuronal resting membrane potentials to at least −50 mV (Bédécarrats et al., 2021). A calcium-free solution (“0 Ca + Co”) in which all calcium (CaCl_2_) was replaced with equimolar (10 mM) cobalt (CoCl_2_) was composed of: (in mM): 450 NaCl, 10 KCl, 30 MgCl_2_, 20 MgSO_4_, 10 CoCl_2_, 10 HEPES, 0.5 EGTA, adjusted to pH 7.5). D-tubocurarine (Merck-Sigma-Aldrich) was used at a concentration of 1 mM in ASW. Tetrodotoxin (TTX, Tocris) was diluted in distilled water from a 0.1 mM stock solution and added to Low Ca + Co saline at a concentration of 1 μM. Tetraethylammonium (TEA, Merck-Sigma-Aldrich) was diluted in Low Ca + Co saline at a concentration of 50 mM. Flufenamic acid (FFA, Merck-Sigma-Aldrich) was first diluted in 100 mM DMSO and then in Low Ca + Co saline at a final concentration of 100 μM (DMSO, 0.1%). Cyclopianozic acid (CPA, Merck-Sigma-Aldrich) was diluted to 50 mM in DMSO and used at a final concentration of 20 μM in Low Ca + Co (DMSO, 0.04%).

### Electrophysiological recordings

Spontaneously expressed cycles of radula motor output (BMPs) were monitored with wire pin extracellular electrodes placed in direct contact with the cut stumps of the protraction I2 (I2 n.), retraction 2,1 (n. 2,1) and radular closure motor (Rn.) nerves. Intracellular electrodes were made from pulled glass capillaries (15–30 MΩ) filled with KCH_3_CO_2_ (2 M). Intracellular signals were amplified with an Axoclamp-2B amplifier (Molecular Devices, Palo Alto, CA), digitized with a CED (Cambridge Electronic Design) interface (sampling rate 5 kHz), then acquired and analyzed with Spike 2 software (Cambridge Electronic Design, UK). B63 interneurons and B31/B32 motoneurons were identified as previously described (Susswein and Byrne, 1988; Nargeot et al., 2009). Relatively brief intracellular current pulses (duration 5 or 8 s) were injected to trigger plateau-like activity in B63 under ASW. These pulse durations correspond approximately to the rising phase durations of plateau-like depolarizations triggered spontaneously by the ongoing intracellular calcium oscillation in B63. It is noteworthy that shorter pulses did not, or were less likely to, elicit plateaus. On the other hand, longer injected pulse durations did not enhance the expression or durations of plateaus because of the onset of the subsequent retraction phase of the BMP, which inhibits B63 activity. After chemical synapse blockade under Low Ca + Co saline, longer pulses of 30 or 60 s, corresponding to the spontaneous depolarization durations of B63 in such a condition, were used.

### Data analysis

The amplitudes of B63’s spontaneous and current pulse-evoked plateau-like potentials were measured from the resting (or pre-pulse) membrane potential until the initial level of step-like, maintained depolarization occurring either spontaneously or after termination of a triggering current pulse. The simultaneous voltage variation in a contralateral B31/B32 neuron, which is postsynaptic to a given B63, was measured using the same criteria. The durations of spontaneous or evoked plateau-like potentials in each recorded B63 and its contralateral B31/B32 partner were measured from the time point of B63’s spontaneous step-like depolarization, or from the end of a triggering current pulse in B63, until the neuron’s spontaneous repolarization to resting membrane potential.

The underlying subthreshold oscillations in membrane potential of B63 neurons were analyzed between a bandwidth of 0.00390 to 0.125 Hz by Fast Fourier Transform (FFT) analysis using R software (R Core Team, 2021). Intracellular voltage recordings were first smoothed with a Spike 2 “Smooth” filter with a time constant of 500 ms to suppress action potentials and down-sampled at 1 Hz to decrease the time of computer processing. The peak magnitudes of spectral densities were determined in 400 s excerpts of B63 intracellular recordings. Sinusoidal waveforms corresponding to plateau-triggering oscillations detected on the FFT periodograms were computed and reconstructed from Wavelet decomposition using the R-CRAN package “WaveletComp” (Roesch and Schmidbauer, 2018).

### Statistical analyses

Two-tailed Mann–Whitney *U* tests were used for unpaired comparisons of two independent groups (U statistic). Comparisons of the proportions of neurons from different preparations expressing evoked plateau-like potentials were made using the two-tailed Fisher’s exact test in unpaired-sample procedures. A Kruskal–Wallis test was used to assess the significance of the overall group differences between >2 independent groups (H statistic). *Post hoc* pairwise comparisons were performed with a Conover’s test with single-step adjustment (q statistic). All statistical tests were conducted with the R-CRAN “Base” and “PMCMRplus” package (Pohlert, 2019) and any comparison differences were considered significant for a probability level of *p* < 0.05. Bars in figures represent Mean ± SEM, *p < 0.05, **p < 0.01, ***p < 0.001, n.s., non-significant.

## Results

### Plateau potentials in B63 can occur independently of B31/B32 plateauing

**FIGURE 1:**
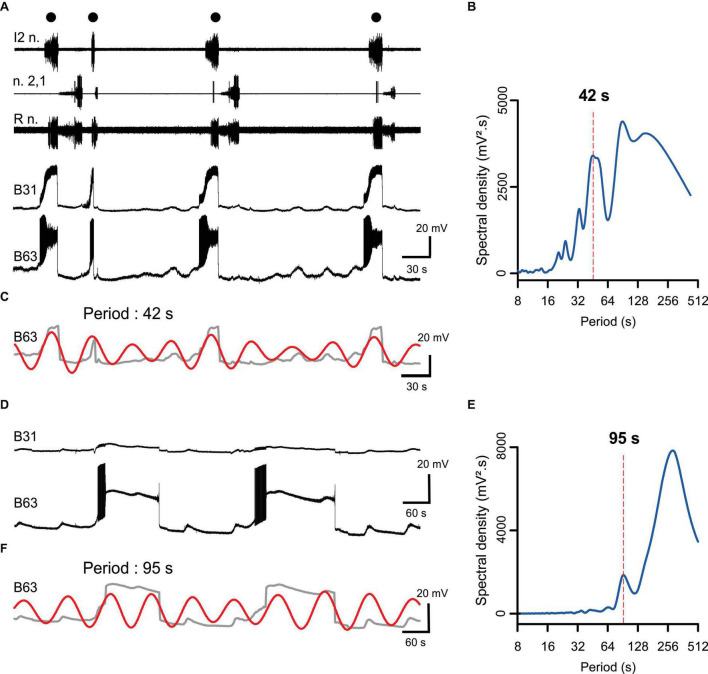
Spontaneous expression of B63 and B31 neuron plateau-like potentials and underlying rhythmic membrane potential oscillations associated with buccal motor pattern (BMP) genesis. **(A)** Simultaneous extracellular recordings from motor nerves of isolated buccal ganglia (top 3 traces) and intracellular recordings of a B63 interneuron and a contralateral B31 protractor motoneuron. Black dots indicate individual spontaneous BMPs, each associated with a large amplitude, prolonged depolarization and burst of action potentials in the recorded B63/B31 neuron pair. **(B)** FFT periodogram of the B63 recording as illustrated in panel **(A)**, indicating an underlying rhythmic fluctuation in the neuron’s membrane potential (dashed line, period 42 s). **(C)** Wavelet-based reconstruction of this fastest (42 s) voltage oscillation (red trace) superimposed on the smoothed B63 membrane voltage trace in panel **(A)** (gray). Each plateau-like potential arises occasionally from the positive peak of an oscillation cycle. Buccal nerve abbreviations: l2n., n.2,1, Rn., are protractor, retractor and closure motor nerves, respectively. **(D)** Intracellular recordings of the same B63 and B31 as in panel **(A)**, during a subsequent perfusion of a Low Ca + Co solution that blocks chemical synaptic connections. Plateau-like depolarizations were still spontaneously expressed in B63 (bottom trace) despite the absence of accompanying B31 bursting. **(E)** FFT periodogram of the B63 recording as illustrated in panel **(D)**, indicating an underlying rhythmic fluctuation in the neuron’s membrane potential (dashed line, period 95 s). **(F)** Wavelet-based reconstruction of this fastest (95 s) voltage oscillation (red trace) superimposed on the smoothed B63 membrane voltage trace from panel **(D)** (gray).

Under normal saline (ASW) conditions, the premotor pacemaker B63 neuron spontaneously expresses endogenous oscillations in membrane potential (Figures 1A–C) that occasionally trigger a large amplitude, plateau-like depolarization and intense burst discharge. This in turn is associated with a plateauing depolarization in B31 neurons and buccal motor pattern (BMP) genesis (Figure 1A). In the condition of chemical synaptic blockade, B63’s membrane potential oscillation and its plateau-like activity persisted, whereas plateauing activity ceased in the B31 neurons (Figures 1D–F). These data therefore suggested that the plateau-like potentials occurring in B63 may arise from membrane property that is intrinsic to pacemaker neuron itself.

**FIGURE 2:**
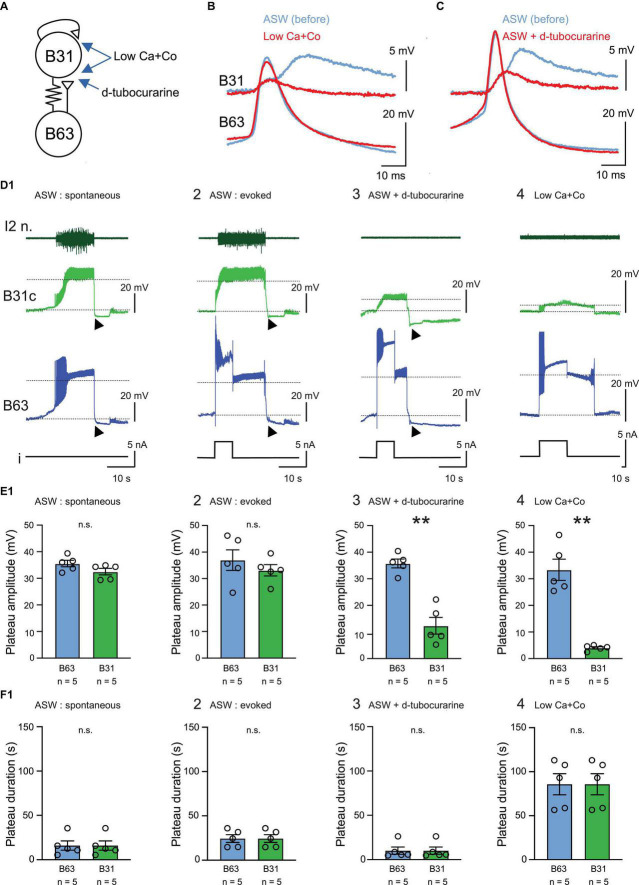
Endogenous plateauing property of the B63 neuron. **(A)** Schematic of the mixed electrical (resistance symbol) and chemical (triangles) synaptic connectivity between the B63 and B31 neurons (circles). The arrows indicate the respective sites of action of Low Ca + Co and d-tubocurarine containing bathing solutions. **(B,C)** Representative paired intracellular recordings of single B63 action potentials and the corresponding post-synaptic potentials in B31/B32, before (blue) and during (red) application of Low Ca + Co perfusion **(B)** or d-tubocurarine (**C**, 1 mM in ASW). **(B,C)** Are from different preparations. **(D)** Simultaneous extracellular recording of the protraction nerve I2 n. and intracellular recordings of spontaneous or evoked activity in a B63 neuron and a contralateral protractor motoneuron B31 in 4 different indicated experimental conditions **(D1–D4)**. In each case, a monitor of intracellular current injected into B63 is shown (i). Dotted lines indicate baseline and depolarized plateau potential levels used for determining both neurons’ plateau amplitudes. Black arrowheads indicate post-plateau synaptic inhibition during the retraction phase of the BMP, which terminates B63 and B31 plateaus in ASW. The recordings in panels **(D1–D3)** are from the same neuron pairs in a single preparation, before **(D1,D2)** and during **(D3)** d-tubocurarine application. Under ASW, spontaneous plateau-like potentials **(D1)** or experimentally evoked potentials **(D2)** are expressed concomitantly in B63 and B31 in association with impulse bursts recorded from the axons of B31 motor neurons in l2n. Such plateau-like depolarizations in B31 and associated axonal discharge in I2n no longer occurred in the presence of d-tubocurarine (1 mM, **D3**) or after complete synaptic isolation of the neurons in Low Ca + Co saline **(D4)**. **(E)** Quantification of plateau depolarization amplitude in B63 and B31 in the 4 experimental conditions illustrated in panel **(D)** [**E1**, *U* = 6, *p* = 0.222; **E2**, *U* = 8, *p* = 0.4206; **E3**, *U* = 0, *p* = 0.008, **E4**, *U* = 0; *p* = 0.008. Between group comparison for B63, *H* = 0.6, *p* = 0.903 (n.s.); for B31, *H* = 16.1, *p* < 0.001 with a significant difference in the d-tubocurarine and Low Ca + Co saline groups compared to each of the other experimental conditions *q* > 4.437, *p* < 0.029]. **(F)** Quantification of plateau depolarization durations in B63 and B31. These durations were identical for both neurons in each of the four experimental conditions (*U* = 12.5, *p* = 1). However, a significant difference was evident between the experimental conditions (*H* = 13.469, *p* < 0.001), with plateau durations being longer in Low Ca + Co saline compared to each of the other saline conditions (*q* > 4.371, *p* < 0.032), no other significant difference was apparent (*q* < 3.985, *p* > 0.054). **p < 0.01.

In a second set of experiments using isolated buccal ganglion preparations, we asked whether the plateau-like depolarizations in B63 can also be expressed under normal ASW in the absence of corresponding B31/B32 neuron activation. The impetus for these experiments was that activity in the B63 neurons was previously thought to drive BMP generation through a combination of monosynaptic fast cholinergic EPSP production in, and electrical coupling with, the contralateral B31/B32 motor neurons (Figures 2A–C; Hurwitz et al., 1997, 2003). In this scheme, when sufficiently depolarized, the B31/B32 cells express a plateau-like state and a prolonged burst of action potentials via self-excitatory synapses, which in turn sustains B63’s depolarization through their electrical coupling (Figures 2D1, E1, F1; Saada et al., 2009).

With sufficient strength, brief pulses (5 or 8 s) of depolarizing current injected into a recorded B63 neuron could trigger long-lasting plateau-like depolarizations of similar amplitude in both the injected B63 itself and a simultaneously recorded B31 neuron (Figures 2D2, E2, F2). Although single current pulses did not always trigger a plateau-like activity in recorded B63/B31 pairs, presumably due to ongoing or elicited synaptic inhibition within the buccal network, the ability for successful pulses to trigger conjoint B63/B31 plateau-like depolarizations was observed in all tested preparations (5/5). Since the B31 neuron’s depolarization is thought to be evoked by fast cholinergic excitation from B63, the nicotinic antagonist D-tubocurarine (1 mM) was applied to test whether the two neuronal responses could be dissociated. The presence of D-tubocurarine in the bath saline effectively blocked the chemical component of EPSPs in B31/B32 elicited by impulses in B63 (Figure 2C). Consequently, the former’s synaptic response to B63 activation was now strongly reduced (Figures 2D3, E3, F3) and was insufficient to reach threshold for impulse firing, as indicated by the total absence of spikes in B31/32’s axons monitored in the I2 motor nerve (Figure 2D3). In contrast, in all preparations (5/5) the initial injected current pulse still elicited a plateau-like depolarization and prolonged burst firing in B63 itself, both of which outlasted the triggering pulse and with a plateau amplitude that remained unchanged from control (compare Figures 2D2, D3).

This dissociation of the membrane behavior of the B63 and B31/32 neurons was further confirmed by exposure to a Low Ca + Co bath solution to block chemical synapses throughout the buccal network (Figure 2B). In this condition, brief current injection into a B63 again elicited a sustained, large amplitude voltage shift but produced only a weak subthreshold depolarization in a simultaneously recorded B31 neuron (Figures 2D4, E4). Presumably, this residual response resulted from the latter’s electrical coupling with B63 (see Figures 2A, B). It is noteworthy, furthermore, that in Low Ca + Co saline, the mean duration of evoked B63 plateaus was significantly increased compared to the duration observed in standard ASW conditions (Figure 2F4; *cf*. Figures 2F1, F2). This increase was an expected consequence of the loss of chemical inhibitory synaptic influences throughout the buccal network, such as those associated with the retraction phase of normal BMP genesis.

Altogether, these results are consistent with the idea that although chemical excitation from B63 acting in combination with B31/B32’s autapses may contribute to plateau-like behavior in these two cell types, this synaptic mechanism is not exclusively responsible for B63’s plateauing capability, but rather, it results at least in part from an endogenous membrane property.

### Ionic mechanisms for B63 plateauing

Plateau potential generation is the expression of a bistable, all-or-none electrical behavior that allows cells to maintain a lasting depolarized membrane potential before switching back to resting potential, either spontaneously or in response to hyperpolarizing synaptic inputs or negative current injection (Russell and Hartline, 1982; Hartline and Graubard, 1992). The depolarized plateau state of this bistability is typically maintained by persistent inward currents through voltage-dependent cationic channels, while plateau termination is due to voltage-induced closure of these channels and/or to outward currents though calcium-gated potassium channels (Golowasch and Marder, 1992; Russo and Hounsgaard, 1996; Tabak et al., 2011).

**FIGURE 3:**
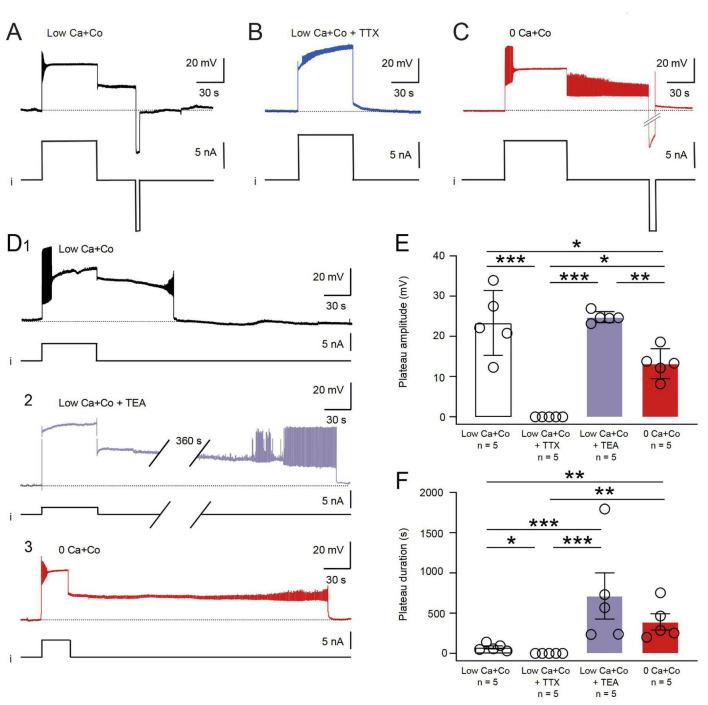
Ion conductances involved in the maintenance and termination of B63 plateau potentials. **(A)** Control: a current pulse-evoked B63 plateau potential in Low Ca + Co saline and its termination by a negative pulse injection (i). **(B)** TTX (1 μM) added to the Low Ca + Co bathing saline prevented the ability of positive current pulse injection (i) to elicit a plateau potential. **(C)** In 0 Ca + Co saline, a plateau potential could still be elicited and terminated by injected positive and negative current pulses, respectively (i). **(D)** Spontaneous plateau termination in Low Ca + Co saline **(D1)** was considerably delayed in the presence of 50 mM TEA (**D2**; the break in the recording trace corresponds to 360 s) or in the complete absence of saline Ca^2+^ **(D3)**. **(E)** The amplitudes of evoked B63 plateaus were significantly different between the experimental conditions [*H* = 15.287, *p* < 0.001: Low Ca + Co versus Low Ca + Co + TTX, *q* = 9.02, *p* < 0.001; versus Low Ca + Co + TEA, *q* = 1.265, *p* = 0.807 (n.s.); versus 0 Ca + Co, *q* = 4.587, *p* = 0.024. Low Ca + Co + TTX versus Low Ca + Co + TEA, *q* = 10.281, *p* < 0.001; versus 0 Ca + Co, *q* = 4.428, *p* = 0.030. Low Ca + Co + TEA versus 0 Ca + Co, *q* = 5.852, *p* = 0.004]. **(F)** Comparison of evoked B63 plateau durations until spontaneous plateau termination. The durations were significantly different between the experimental conditions [*H* = 16.389, *p* < 0.001: Low Ca + Co versus Low Ca + Co + TTX, *q* = 4.71, *p* = 0.020; versus Low Ca + Co + TEA, *q* = 7.542, *p* < 0.001; versus 0 Ca + Co, *q* = 6.600, *p* = 0.001. Low Ca + Co + TTX versus Low Ca + Co + TEA, *q* = 12.256, *p* < 0.001; versus 0 Ca + Co, *q* = 11.314, *p* < 0.001. Low Ca + Co + TEA versus 0 Ca + Co, *q* = 0.942, *p* = 0.908 (n.s.)]. *p < 0.05, **p < 0.01, ***p < 0.001.

Under chemical synaptic blockade with Low Ca + Co exposure, the B63 neurons continued to express such bistable membrane behavior, with a prolonged plateau state that could be triggered by depolarizing current pulse injection and terminated prematurely by hyperpolarizing current injection (Figure 3A). To investigate the contribution of Na^+^ and/or Ca^2+^ ions to the onset and maintenance of B63’s active depolarized state, experiments (*n* = 5) were conducted in which the sodium channel blocker TTX (1 μM) was added to the Low Ca + Co bathing solution. In all preparations under this condition, injected depolarizing current pulses failed to trigger B63 plateau potentials (Figures 3B–F). In contrast, in a separate group of preparations (*n* = 5) that were bathed in 0 Ca + Co saline, B63 was still able to express sustained plateau potentials in response to positive current pulse injection (5/5 preparations), and then terminated spontaneously or in response to brief hyperpolarizing current pulses (Figures 3C, D3). On this basis, therefore, extracellular Na^+^ influx through TTX-sensitive membrane channels, but not extracellular Ca^2+^ influx, appears to be necessary for the expression of B63’s endogenous plateau property. Nevertheless, the amplitude of B63 plateaus was significantly reduced under 0 Ca + Co compared to Low Ca + Co, thereby suggesting that, although Ca^2+^ is not necessary for the expression of a mainly Na-dependent mechanism, calcium influx nonetheless contributes partly to B63’s plateau depolarization (Figure 3E).

In normal saline (ASW) conditions, the spontaneous termination of individual plateaus and associated impulse bursts in B63, as in other buccal neurons generating the protractor phase of each BMP, is triggered by an inhibitory synaptic drive from neurons active during the retraction phase of each radula bite cycle (Hurwitz and Susswein, 1996; Sasaki et al., 2013). Under Low Ca + Co or 0 Ca + Co solutions, which suppress these inhibitory influences, B63 expressed greatly extended plateau potentials that eventually terminated spontaneously (Figures 3D1, D3, E, F). This step-change cessation could also be prematurely induced by the intracellular injection of a hyperpolarizing current (see Figures 3A, C), thereby suggesting the contribution of a voltage-dependent mechanism.

To test for a probable contribution of potassium channels in B63’s plateau termination, preparations were superfused with or without the potassium channel blocker TEA (50 mM) in Low Ca + Co saline. Under this condition, plateaus were still able to be evoked in 5/5 preparations. In the presence of TEA (*n* = 5), as compared to in the blocker’s absence (*n* = 5), the durations of B63’s plateau potentials were considerably and significantly prolonged (Figure 3D2, compare with Figures 3D1, E). Plateau durations were also considerably modified by changes in the concentration of calcium ions, with plateaus lasting several tens of seconds in the presence of Ca^2+^ (3 mM in the Low Ca + Co solution, *n* = 5), but were maintained for hundreds of seconds in the cation’s absence (0 Ca + Co solution, *n* = 5) (Figure 3D3, compare with Figures 3D1, F). These findings thus lead to the conclusion that a voltage-dependent potassium current regulated by calcium contribute to B63’s plateau termination.

### Different cation channels underlie B63’s endogenous plateau and oscillatory properties

**FIGURE 4:**
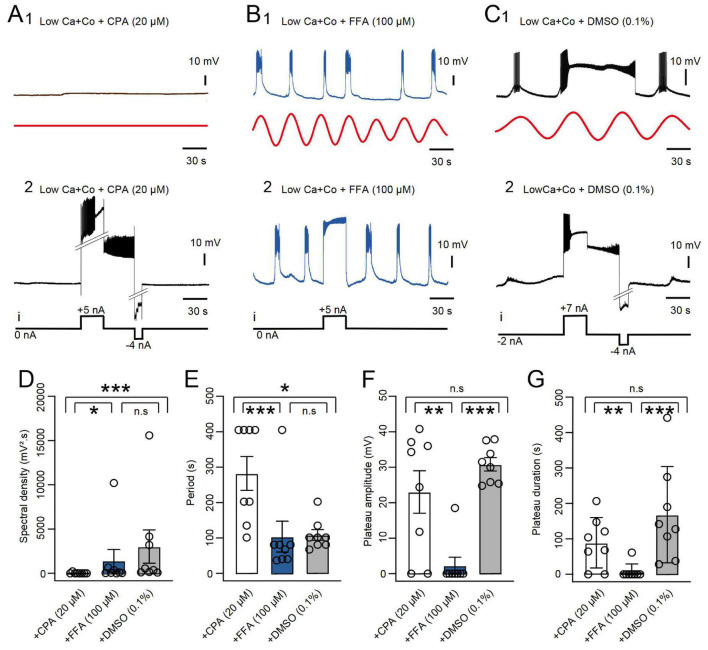
Differential sensitivity of B63 plateauing and oscillation to CPA and FFA. **(A)** Effect of CPA. The presence of the SERCA blocker (20 μM in Low Ca + Co) suppressed a recorded B63’s spontaneous membrane potential oscillation (**A1**, black trace, raw recording; red trace, reconstructed waveform from the peak FFT spectral density), but not the ability of injected current pulses (i) to trigger (+5 nA) and terminate (–4 nA) a plateau potential **(A2)**. **(B)** The presence of the non-specific cation channel blocker FFA (0.1 mM in Low Ca + Co saline) did not suppress the spontaneous membrane potential oscillation and associated short-lasting bursting activity in a B63 neuron (**B1**, red trace: reconstructed waveform from the peak FFT spectral density), but it prevented expression of spontaneous or current pulse-evoked, long-lasting plateau potentials **(B2)**. **(C)** The vehicle DMSO alone (0.1% in Low Ca + Co saline) did not affect B63’s capability to produce spontaneous membrane potential oscillations (**C1**, red trace: reconstructed waveform from the peak FFT spectral density), spontaneous, long-lasting plateau potentials, or their triggering on or off by positive (+7 nA) and negative (–4 nA) current pulses, respectively **(C2)**. Note that in panel **(C2)**, the B63 cell was held continuously hyperpolarized with constant current (–2 nA) to prevent spontaneous plateau generation. **(D)** Comparison of the amplitudes of spontaneous oscillation (FFT spectral density) in the three pharmacological conditions (*n* = 8 in each case). The oscillation amplitude was significantly different between groups [*H* = 11.945, *p* < 0.001: Low Ca + Co + CPA versus Low Ca + Co + FFA, *q* = 4.065, *p* = 0.0236; versus Low Ca + Co + DMSO, *q* = 6.6845, *p* < 0.001. Low Ca + Co + FFA versus Low Ca + Co + DMSO, *q* = 1.344. *p* = 0.179, (n.s.)]. **(E)** Comparison of oscillation periods in the three pharmacological conditions. The period was significantly different between groups [*H* = 11.968, *p* < 0.001: Low Ca + Co + CPA versus Low Ca + Co + FFA, *q* = 6.671, *p* < 0.001; versus Low Ca + Co + DMSO, *q* = 4.226, *p* = 0.018. Low Ca + Co + FFA versus Low Ca + Co + DMSO (*q* = 2.444, *p* = 0.218 (n.s.)]. **(F)** Comparison of B63 plateau amplitudes in the three pharmacological conditions (*n* = 8 in each case). The amplitude was significantly different between groups [*H* = 12.175, *p* < 0.001: Low Ca + Co + CPA versus Low Ca + Co + FFA *q* = 5,007, *p* = 0.005; versus Low Ca + Co + DMSO, *q* = 1.574, *p* = 0.517 (n.s.); Low Ca + Co + FFA versus Low Ca + Co + DMSO, *q* = 6.581, *p* < 0.001]. **(G)** Comparison of plateau durations in the three pharmacological conditions (*n* = 8 in each case). The duration was significantly different between groups [*H* = 12.381, *p* < 0.001: Low Ca + Co + CPA versus Low Ca + Co + FFA, *q* = 4.622, *p* = 0.010; versus Low Ca + Co + DMSO, *q* = 2.239, *p* = 0.270 (n.s.). Low Ca + Co + FFA versus Low Ca + Co + DMSO, *q* = 6.861, *p* < 0.001]. *p < 0.05, **p < 0.01, ***p < 0.001.

In normal ASW conditions, plateau potentials in the B63 neuron are triggered spontaneously by an ongoing low amplitude oscillation in the interneuron’s membrane potential (see Figure 1) that arises from organelle-derived fluxes in intracellular calcium (Bédécarrats et al., 2021). Both the plateaus (as described above) and their underlying voltage oscillation are blocked by TTX, indicating in each case, a contribution of sodium and/or non-selective cation membrane channels (see Figure 3B and Bédécarrats et al., 2021). To determine whether the same or different membrane channels are implicated in the expression of these endogenous properties, the specific effects of two different pharmacological agents was tested. Firstly, the membrane potential oscillation of B63 was suppressed by the presence of reticulum endoplasmic calcium pump inhibitor CPA (20 μM in a Low Ca + Co solution; Figures 4A1, D, E; see also Bédécarrats et al., 2021), and consequently, also prevented the spontaneous expression of plateau potentials (*n* = 8; Figure 4A1, compare with Figure 4C1). Nonetheless, in the majority of these preparations (6/8), B63 neurons were still able to produce plateau potentials in response to depolarizing current injection (Figure 4A2, compare with Figures 4C2, F, G). This proportion of preparations in which B63 still expressed current pulse-evoked plateaus was not significantly different from that found in ASW (5/5, *p* = 0.487), in Low Ca + Co (5/5, *p* = 0.487) or in Low Ca + Co + DMSO (8/8, *p* = 0.467) thereby indicating that CPA did not in fact suppress this membrane property. In contrast, the non-selective cation channel blocker FFA (0.1 mM in a Low Ca + Co solution, *n* = 8) did not block B63’s spontaneous membrane oscillation (Figures 4B1, D, E), which led to action potential bursts, but solely with short durations, on nearly every cycle. Moreover, in almost all of these tested preparations (7/8), experimental depolarization by current pulse injection, also failed to trigger any sustained plateau-like potentials (Figures 4B2, D). This proportion of preparations was now significantly different from that in ASW (0/5, *p* = 0.003), in Low Ca + Co (0/5, *p* = 0.003) or in Low Ca + Co + DMSO (0/8, *p* = 0.0). In addition, quantitative comparisons between the amplitude and cycle period of B63’s spontaneous oscillations and of the amplitude and duration of evoked B63 plateaus further indicated that these membrane properties are differently affected by FFA and CPA (Figures 4D–G). Therefore, although the B63 neuron’s oscillatory and plateauing capability both depend on TTX-sensitive membrane channels, the above pharmacological results indicated the involvement of two distinct types of sodium channels.

## Discussion

The genesis of impulse bursting in central neuronal networks plays a fundamental role in decision-making processes and motor pattern genesis. In *Aplysia*, the decision-making process leading to buccal motor cycle production depends on autonomous depolarizations and associated burst activity generated by a small subgroup of network neurons (including B63, B31/B32) that are interconnected by their electrical and chemical synapses (Susswein et al., 2002; Dembrow et al., 2004; Saada et al., 2009; Saada-Madar et al., 2012). In a two-neuron process, individual synapse-driven depolarizations of the B31/B32 motoneurons by the B63 pacemaker neuron become long-lasting, thought to be due to voltage-activated muscarinic autapses that produce a plateau-like activation of these cells, which in turn provide sustaining feedback excitation to B63. The present study extends on this synapse-mediated mechanism by showing that the B63 neuron can express plateau potential generation in the absence of B31/B32 activity. Thus, in addition to an underlying synaptic mechanism, the production of plateau-like potentials in the B63/B31/B32 subset is also likely to result, at least in part, from an endogenous membrane property of B63 itself. This is especially relevant since this pacemaker cell type also possesses an endogenous oscillatory property that can spontaneously initiate plateau potential production (see Figure 1; Bédécarrats et al., 2021). Thus, the decision-making process triggering each radula bite cycle in *Aplysia* evidently relies on a complex interplay between different intrinsic membrane properties and reciprocal electrical and chemical synapses amongst a subset of buccal network neurons.

Neuronal endogenous plateau properties derive from ionic transmembrane channels mediating persistent inward currents that can be triggered by brief synaptic inputs or spontaneous variations in membrane potential (Russell and Hartline, 1978). The onset and maintenance of these plateau potentials have been previously found to depend on voltage-dependent L-type calcium channels (Svirskis and Hounsgaard, 1997), voltage-dependent sodium channels responsible for *I*_NaP_ (Crill, 1996; Elson and Selverston, 1997), non-voltage dependent, calcium activated non-specific cationic channels (CAN) (Partridge and Swandulla, 1988; Zhang et al., 1995; Morisset and Nagy, 1999) or an interplay between these cationic currents (Bouhadfane et al., 2013). B63’s spontaneous membrane potential oscillation that triggers plateau potentials was previously found to arise from an intracellular dynamic involving organelle calcium release (Bédécarrats et al., 2021). The present study now shows that these two intrinsic capabilities rely on different sets of conductances, with distinct electrical and pharmacological characteristics. Although both properties are TTX-sensitive, B63’s oscillation depends on a voltage-independent mechanism that is blocked by a reticulum calcium pump inhibitor (CPA) and is insensitive to the CAN channel blocker FFA (Bédécarrats et al., 2021). In contrast, B63’s plateauing ability which depends on active, voltage-dependent rising and falling phases, can be triggered by a transient depolarization and hyperpolarization, respectively, and is not sensitive to CPA but is so to FFA. It is noteworthy that under FFA, however, the rising phase of each plateau is preserved, resulting in brief impulse burst firing, but a prolonged (lasting for 10s of secs to mins) depolarization indicative of a plateau and accompanying sustained discharge now fails to occur. This is therefore consistent with the likelihood that different ion channels are implicated in the onset and maintenance phases of the plateau. While voltage-dependent channels insensitive to FFA and conveying a persistent Na^+^ current (*I*_NaP_) could contribute to the rising phase, a CAN current (I_CAN_) that is sensitive to FFA may contribute to plateau maintenance. This in turn raises a possible contribution by intracellular calcium stores (the plateau persists in the presence of CPA, which increases the intracellular calcium concentration) or a voltage-dependent process. Such a mechanism was previously described for non-selective cationic channels contributing to stimulus afterdischarge in *Aplysia* bag cells (Wilson et al., 1996). Finally, plateau termination in B63 was found to be sensitive to transient membrane hyperpolarization, the K^+^ channel blocker TEA, and to Ca^2+^ removal, all consistent with the involvement of a voltage- and calcium-activated potassium channel (Golowasch and Marder, 1992; Brezina and Weiss, 1995; Russo and Hounsgaard, 1996). Further characterization of these conductances and their interaction with those underlying B63’s oscillatory capability will thereby increase our understanding of the autonomous decision-making processes for *Aplysia’s* goal-directed actions.

## Data availability statement

The raw data supporting the conclusions of this article will be made available by the authors, without undue reservation.

## Ethics statement

Ethical review and approval were not required for this animal study in accordance with local legislation and institutional requirements.

## Author contributions

AB and RN designed and performed the experiments. AB performed the data analysis, made the initial version of the figures, and wrote the first draft of the manuscript. JS and RN prepared and edited the final version of the manuscript. All authors contributed to the article and approved the submitted version.
